# Characterization of an intertidal zone metagenome oligoribonuclease and the role of the intermolecular disulfide bond for homodimer formation and nuclease activity

**DOI:** 10.1002/2211-5463.12720

**Published:** 2019-08-31

**Authors:** Yvonne Piotrowski, Kristel Berg, David Paul Klebl, Ingar Leiros, Atle Noralf Larsen

**Affiliations:** ^1^ Department of Chemistry Faculty of Science and Technology SIVA Innovation Centre UiT – The Arctic University of Norway Tromsø Norway; ^2^ Present address: School of Biomedical Sciences Faculty of Biological Sciences Astbury Centre for Structural and Molecular Biology University of Leeds Leeds LS2 9JT UK

**Keywords:** crystal structure, homodimer, metagenome, nuclease activity, oligoribonuclease, RNA

## Abstract

The gene encoding MG Orn has been identified from a metagenomic library created from the intertidal zone in Svalbard and encodes a protein of 184 amino acid residues. The *mg orn* gene has been cloned, recombinantly expressed in *Escherichia coli*, and purified to homogeneity. Biochemical characterization of the enzyme showed that it efficiently degrades short RNA oligonucleotide substrates of 2mer to 10mer of length and has an absolute requirement for divalent cations for optimal activity. The enzyme is more heat‐labile than its counterpart from *E. coli* and exists as a homodimer in solution. The crystal structure of the enzyme has been determined to a resolution of 3.15 Å, indicating an important role of a disulfide bridge for the homodimer formation and as such for the function of MG Orn. Substitution of the Cys110 residue with either Gly or Ala hampered the dimer formation and severely affected the enzyme's ability to act on RNA. A conserved loop containing His128‐Tyr129‐Arg130 in the neighboring monomer is probably involved in efficient binding and processing of longer RNA substrates than diribonucleotides.

**Abbreviations**
CMP: cytidine 5′‐monophosphate
MBP: maltose‐binding protein
OD600: optical density at 600 nm
PAA: polyacrylamide
PDB: Protein Data Bank
pNP‐TMP: *p*‐nitrophenyl ester of thymidine 5′‐monophosphate
TEV: tobacco etch virus

Oligoribonuclease (Orn) is assumed to originate from eukaryota and is present in almost all eukaryotes 1, 2. In bacteria, and based on sequenced bacterial genomes, *orn* is mainly distributed in beta‐ and gammaproteobacteria and firmicutes 1. Orn is a processive 3′–5′ exonuclease that converts small oligoribonucleotides to monoribonucleotides and is important for mRNA decay in cells 3. Studies in *Escherichia coli* show that Orn is essential for the viability of the bacteria 3, while *Pseudomonas aeruginosa* cells remain viable in the absence of Orn 4. Interestingly, the human Orn homologue is able to degrade both small single‐stranded RNA and DNA molecules *in vitro* and the authors suggest a role of human Orn in cellular nucleotide recycling 5.

In recent years, several studies of Orn in *P. aeruginosa* have broadened the view on the role(s) of Orn in bacteria. Depletion of Orn leads to accumulation of small RNA molecules in cells, and these can serve as primers for transcription initiation and lead to global alterations in gene expression 6. Orn is also demonstrated to play a central role in intracellular turnover of the bacterial second messenger cyclic‐di‐GMP with implications for bacterial motility, virulence, and biofilm formation 7, 8. A recent study showed that an *orn* mutant of *P. aeruginosa* displayed reduced cytotoxicity mainly by affecting the type III secretion system, further indicating an important role of Orn in bacterial pathogenesis 9. Furthermore, Chen and coworkers showed that a Δ*orn* mutant became highly susceptible to the antibiotic ciprofloxacin, indicating a novel role in antibacterial drug resistance 10.

Orn is a member of the DEDDh superfamily of exoribonucleases and contains four sequence motifs unique to oligoribonucleases 2. It is a small protein of approximately 20 kDa and requires divalent cations for nuclease activity, preferably Mn^2+^ 5, 11, 12. The *E. coli* enzyme exists as a homodimer in solution 12, 13. Through gel filtration experiments, the human homologue of *E. coli* Orn is in one study shown to be a tetramer in solution 5, whereas another study indicates the enzyme to be a homodimer 12. The *E. coli* enzyme is characterized as heat‐stable, has a half‐life of 60 min at 65 °C, and still has residual activity after 10‐min incubation at 100 °C 11. The human enzyme is also quite thermostable and has a temperature optimum for nuclease activity around 50 °C 5. Datta and Niyogi 14 showed that *E. coli* Orn has a higher affinity for longer chain substrates than smaller substrates, but the reaction rate was inversely proportional to the length of the chain. The nuclease activity of the human Orn homologue is also inversely proportional to the length of the single‐stranded substrate 5. Analysis of the kinetic data of human Orn indicates similar *K*_m_ values for short single‐stranded RNA and DNA but degrades short RNA about fourfold more efficiently than ssDNA 5.

Crystal structures of Orn show that they are closely related and topologically arranged into an α + β fold containing 5–6 β‐strands and 9–10 α‐helices (PDB 2GBZ: *Xanthomonas campestris*, PDB 1J9A: *Haemophilus influenzae*, PDB 2IGI: *E. coli*, PDB 3TR8: *Coxiella burnetii*, PDB 5CY4: *Acinetobacter baumannii*). Despite several deposited Orn structures, it is yet unclear how Orn achieves the apparent processive oligoribonucleotide cleaving mechanism, but formation of a stable homodimer is indicated to be important 15. In the *X. campestris* Orn structure (PDB 2GBZ), it is shown that Orn forms a dimer in the crystal through crystallographic symmetry. From the structural analysis, it was shown that hydrophobic interactions as well as several hydrogen bonds (H‐bonds), salt bridges, and a disulfide bond contribute to the formation of a stable homodimer. A very recent publication showing among others an Orn with two uridine molecules bound in the RNA substrate binding site also provides further evidence that hydrophobic interactions, salt bridges, and H‐bonds are important for dimer formation 16.

In this study, we have recombinantly produced, characterized, and determined the three‐dimensional crystal structure of an arctic marine oligoribonuclease, named MG Orn. We further wanted to investigate the role of the intramolecular disulfide bond connecting the two MG Orn monomers, and our results suggest that this disulfide bond is essential for the formation of a functional homodimer and therefore also the ability of the enzyme to degrade small oligoribonucleotides. We also report the ability of MG Orn to act on longer RNA molecules. Finally, we indicate the involvement of a conserved His‐Tyr‐Arg loop in the neighboring monomer in binding of these longer (up to 10mer) RNA substrates.

## Results

The metagenomic oligoribonuclease (MG Orn) described in this paper consists of 184 amino acid residues. The protein has been recombinantly produced with an N‐terminal His_6_‐MBP‐tag followed by a cleavage site for the tobacco etch virus (TEV) protease. After hydrolytic removal of the N‐terminal tag, four amino acid residues (Gly‐Ser‐Phe‐Thr) remain at the N terminus of MG Orn due to the recognition site of the protease. Numbering of the amino acid residues within this paper will be according to the protein sequence of MG Orn, that is, excluding the additional amino acid residues of the tag‐removal reaction.

### Phylogenetic analysis/sequence analysis

**Figure 1:**
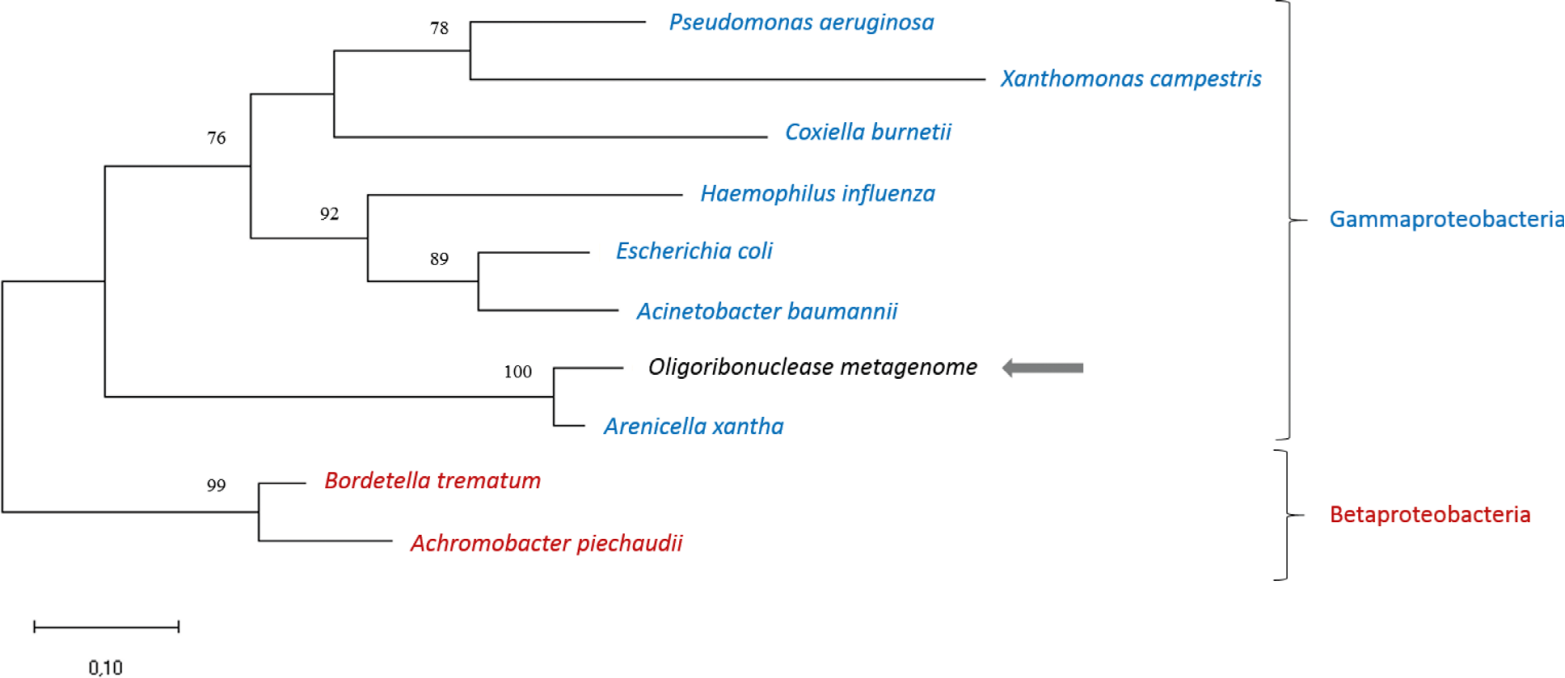
Phylogenetic relationship of MG Orn protein with selected Orn homologues from gamma‐ and betaproteobacteria. Node numbers indicate bootstrap support values, with only values above 50 shown. The investigated metagenome sequence is marked with a gray arrow. The tree is drawn to scale, with branch lengths measured in the number of substitutions per site. The sequences were obtained from GenBank with the following accession numbers: WP_113955167.1 (*Arenicella xantha*), WP_011037314.1 (*Xanthomonas campestris*), RQB22498.1 (*Pseudomonas aeruginosa*), WP_005770781.1 (*Coxiella burnetii*), SST03775.1 (*Acinetobacter baumannii*), WP_021035403.1 (*Haemophilus influenzae*), WP_042004351.1 (*Escherichia coli*), WP_025512385.1 (*Bordetella trematum*), and WP_006218241.1 (*Achromobacter piechaudii*).

A phylogenetic tree based on the maximum likelihood method places MG Orn and the close homologue *Arenicella xantha* Orn in a distinct clade from the other Orn homologues (Fig. 1), as expected from the high sequence identity compared to other Orn homologues (94% versus 50–60%). These two homologues branch out early, just after the shared common gammaproteobacteria ancestor, but their origin is a rather recent event. The statistical bootstrap support value of 100 strongly indicates that MG Orn originates from a species within the *Arenicellales* order, possibly *Arenicellas* or another close relative.

### Biochemical/biophysical characterization

**Figure 2:**
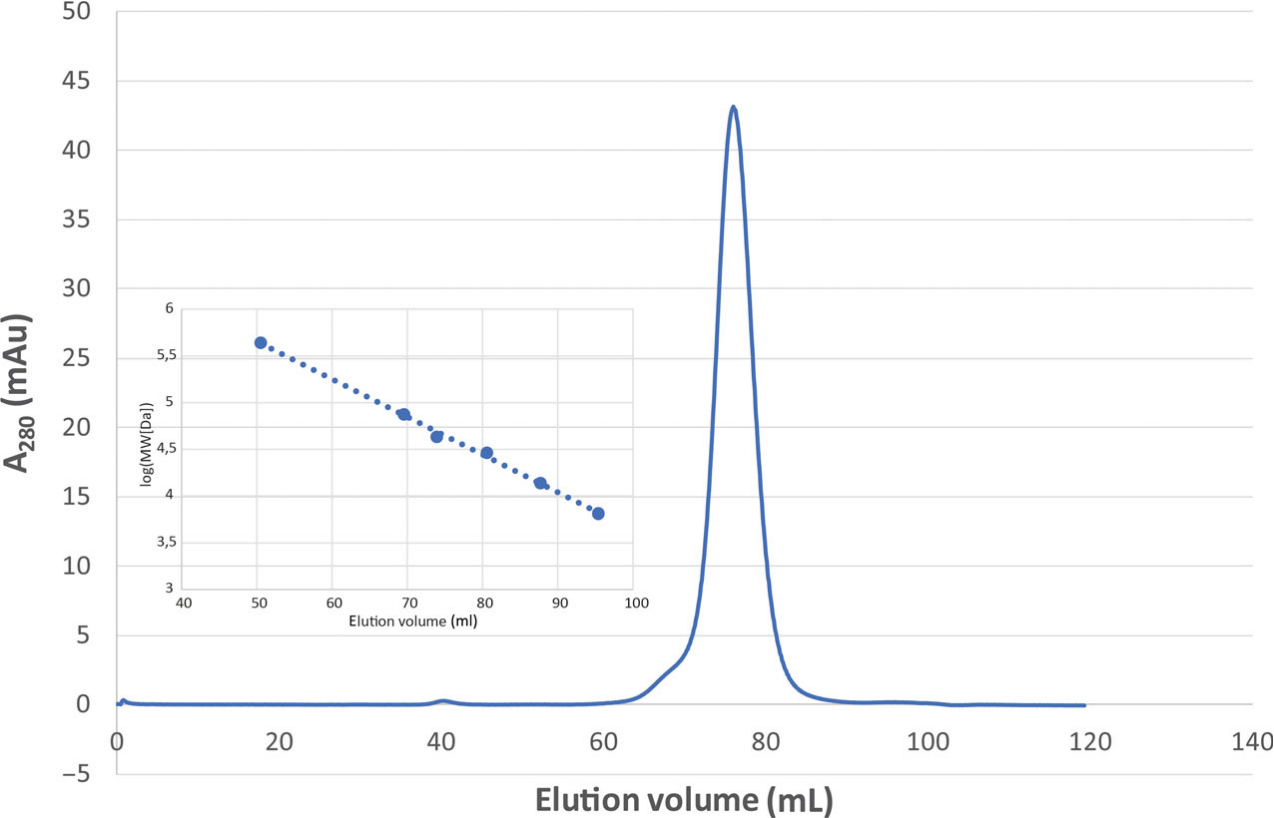
Size‐exclusion chromatography of MG Orn. The inset shows the calibration curve established with Ferritin (440 kDa), Conalbumin (75 kDa), Ovalbumin (43 kDa), Carbonic Anhydrase (29 kDa), RNase A (14 kDa), and Aprotinin (6.5 kDa). The *R* ^2^ value of the regression line is 0.997. Based on the calibration curve and the elution volume, the estimated size of MG Orn is 41 kDa.

**Figure 3:**
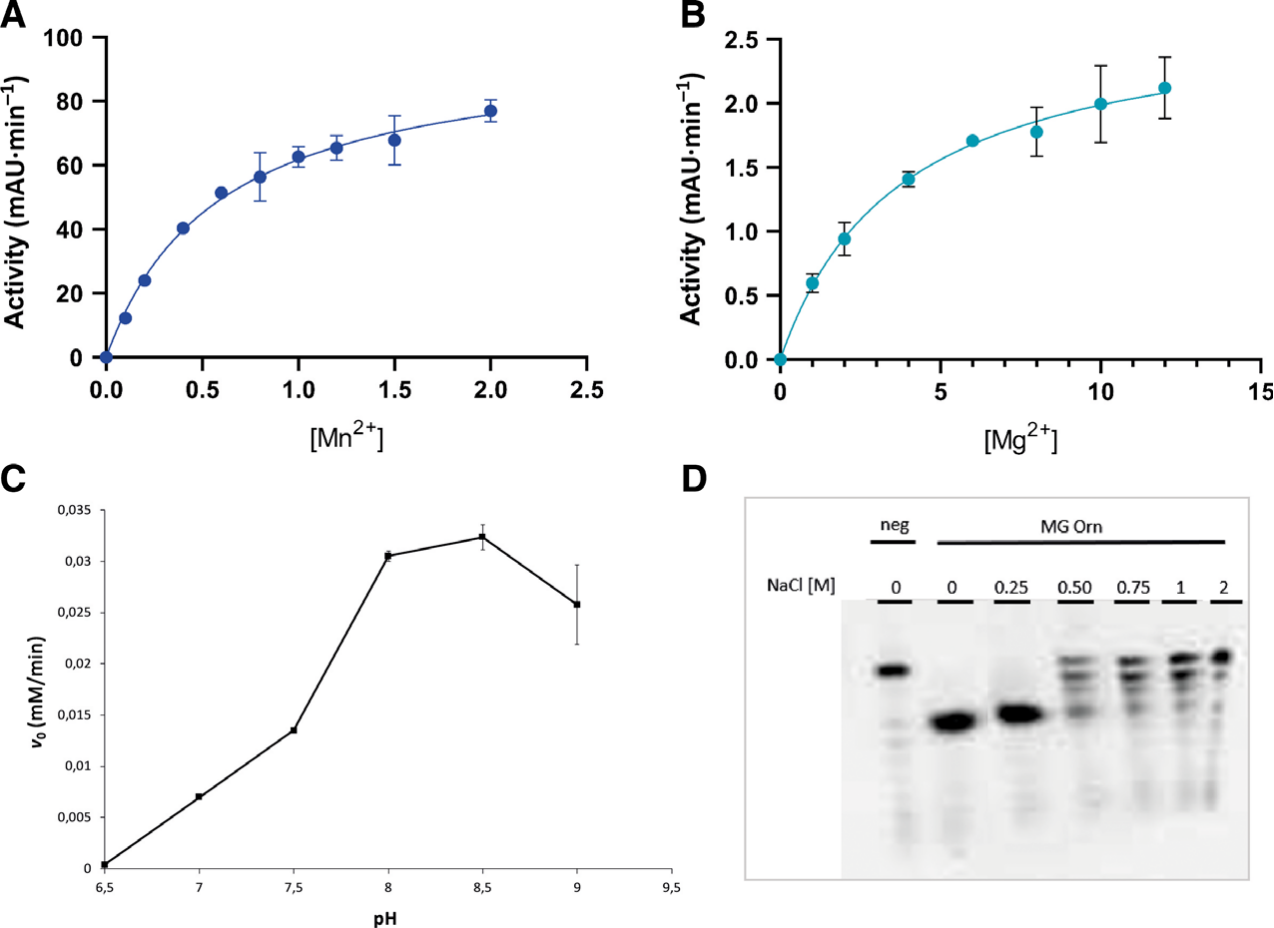
Effect of Mn^2+^ (A) and Mg^2+^ (B), pH (C), and NaCl (D) on the nuclease activity of MG Orn. The effect of the metal ions and pH on the enzyme activity has been determined using the time‐resolved *p*NP‐TMP activity assay at 25 °C as described in Methods with 1.1 μg Orn and varying amounts of Mn^2+^ and Mg^2+^ as well as 1.5 μg MG Orn and pH values from 6.5 to 9 using MES (pH 6.5), HEPES (pH 7–7.5), and Tris (pH 8–9) as indicated in A–C. The rate of hydrolysis of *p*NP‐TMP at the varying pH values was calculated according to Hamdan *et al*. 34. Error bars indicate the standard deviation of the measurements. The effect of NaCl in a range of 0–2 m is shown in (D) and has been tested at 25 °C with the gel‐based nuclease activity assay with 0.05 μm 7mer RNA substrate and 0.8 μg MG Orn in 50 mm Tris‐HCl pH 8.0, 0.2 mg·mL^−1^ BSA, 2% glycerol and 1 mm MnCl_2_ for 15 min. Samples were analyzed on 20% denaturing PAA gels (8 × 8 cm). Reaction buffer was used as negative control (Neg) instead of protein solution.

Size‐exclusion experiments were performed to investigate whether MG Orn was monomeric or dimeric in solution. MG Orn eluted as a single peak corresponding to a protein with a molecular weight of 41 kDa (Fig. 2), clearly indicating that MG Orn existed as a dimer in solution. The effect of divalent cations (Mg^2+^/Mn^2+^) and pH on the nuclease activity of MG Orn has been determined using the *p*NP‐TMP activity assay (see Methods). MG Orn showed an absolute requirement for a divalent metal ion, with Mn^2+^ being clearly preferred over Mg^2+^ (Fig. 3A,B). Furthermore, MG Orn possessed a quite narrow pH range for optimal activity of pH 8–9 with an apparent optimum at pH 8.5 (Fig. 3C).

The effect of various salt concentrations on the nuclease activity of MG Orn was analyzed using the gel‐based nuclease activity assay. MG Orn showed significant salt (NaCl) tolerance using 7mer RNA as substrate, and robust hydrolytic activity was observed in the presence of up to 250 mm NaCl. There is still some residual activity observed in the presence of 500 mm NaCl with activity gradually declining up to 2 m NaCl (Fig. 3D). Using the dinucleotide analogue *p*NP‐TMP as substrate (*p*NP‐TMP assay), MG Orn possessed even higher salt tolerance and little effect on nuclease activity was observed even at 2 m NaCl (results not shown).

**Figure 4:**
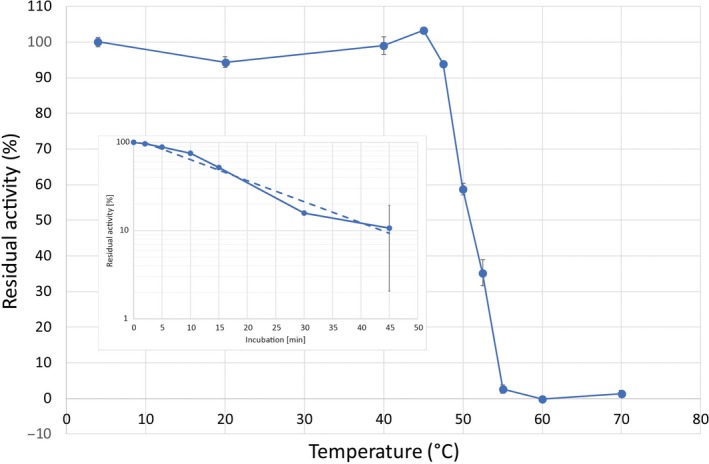
Temperature stability profile of MG Orn. The enzyme was preincubated at the respective temperature for 15 min and subsequently tested with the time‐resolved *p*NP‐TMP assay at 25 °C in 50 mm Tris pH 8.0, 200 mm NaCl, 1 mm MnCl_2_, 1.5 mm *p*NP‐TMP, and 1.5 μg MG Orn. Activity measured of the sample preincubated at 4 °C was set as 100% residual activity. Error bars indicate the standard deviation of the measurements. The inset shows the graph determining the half‐life of the enzyme at 50 °C.

To assess the thermal stability of MG Orn, the enzyme was preincubated at different temperatures for 15 min and residual activity was measured using the *p*NP‐TMP activity assay. MG Orn was rapidly inactivated at temperatures above 48 °C, with a half‐life of about 15 min at 50 °C (Fig. 4).

**Figure 5:**
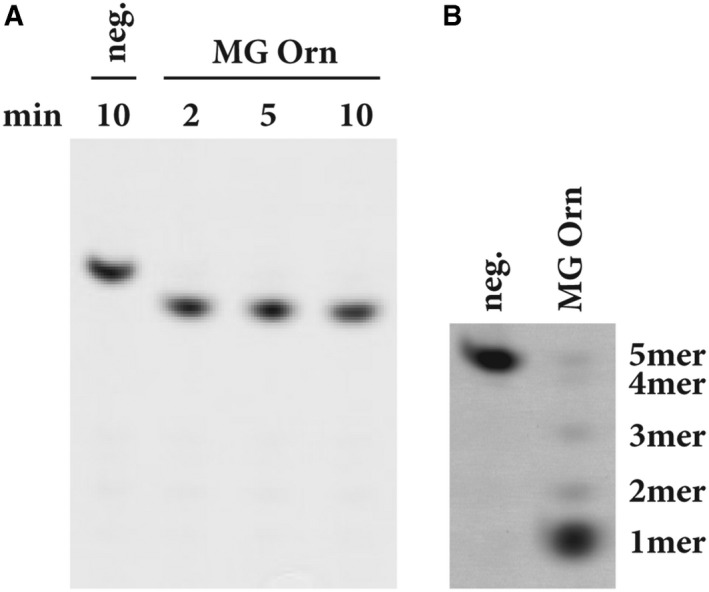
(A) Directionality of MG Orn. Reactions have been performed with the enzyme assay for determination of directionality at 25 °C in 50 mm Tris pH 8.0, 150 mm NaCl, 1 mm MnCl_2_, 1 mm DTT, 0.2 mg·mL^−1^ BSA, and 2% glycerol with 25 nm 7mer‐62OMe RNA substrate and 0.74 μg MG Orn. Samples have been taken at several points in time as indicated. (B) Degradation of 5′ FAM‐labeled 5mer RNA substrate at 25 °C. Reactions have been performed with the gel‐based nuclease activity assay with 100 nm substrate and 40 ng MG Orn in 50 mm Tris pH 8.0, 200 mm NaCl, 1 mm MnCl_2_. After 5 min, the samples were collected and analyzed on a 20% denaturing PAA gel (40 × 20 cm). ‘Neg.’ indicates the 5′ FAM‐labeled 5mer RNA substrate with no enzyme added to the reaction.

The 7mer RNA substrate 7mer‐62OMe (5′‐[FAM]CCCCC[mC]C‐3′) was used to investigate the directionality of MG Orn. The substrate contains a methyl group at the 2′ hydroxyl of the ribose at C^6^. This 2′‐O‐methylation blocks ribonuclease function. Nuclease activity proceeding in 3′–5′ direction will result in one 6mer RNA with the fluorophore FAM linked to the 5′ end and one unlabeled cytidine 5′‐monophosphate (CMP). If the nuclease proceeds in 5′–3′ direction, the substrate will be cleaved into one FAM‐labeled CMP, four unlabeled CMPs, and one unlabeled CDP. MG Orn proceeds in 3′–5′ direction as in all reactions a band just below the RNA substrate (7mer with 2′‐O‐Me) can be detected, indicating a FAM‐labeled 6mer RNA (Fig. 5A). Using 5′ FAM‐labeled 5mer RNA as substrate, MG Orn effectively degraded the substrate to monoribonucleotide products, further proving its 3′–5′ directionality (Fig. 5B). MG Orn was also able to degrade short single‐stranded DNA (5mer and 10mer) although with much lower efficacy than with RNA (results not shown).

### Structural analysis

**Figure 6:**
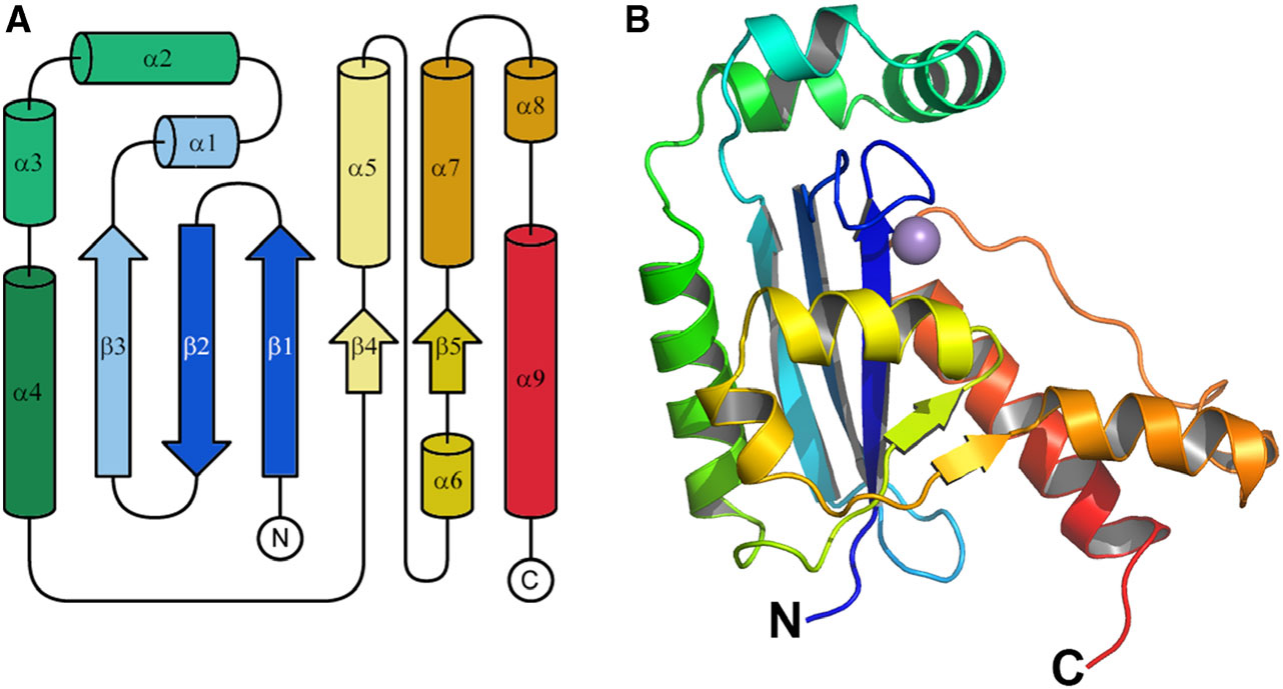
Topology diagram and monomeric structure of MG Orn. (A) Topology diagram displaying the order of the secondary structure elements. (B) Cartoon representation of a monomer of MG Orn. In both figures, the N and C termini are indicated, and secondary structure elements are colored in rainbow colors ranging from blue to red. The bound Mn^2+^ ion is shown as purple sphere.

The crystal structure of MG Orn was determined at 3.15 Å resolution, by the molecular replacement method, using the oligoribonuclease from *X. campestris* (PDB: 2GBZ) as a template. A summary of the data collection, refinement, and validation statistics is given in Table 1. The crystal structure of MG Orn contains three monomers in the asymmetric unit. For all three chains, a continuous polypeptide comprising amino acid residues 5–181 (chains A and B) and 5–182 (chain C), respectively, was visible in electron density and included in the final model. The three monomers were tightly restrained in refinement and are thus virtually identical with an r.m.s. deviation of 0.001 Å. Each of the three molecules in the asymmetric unit consists of nine α‐helices and five β‐strands in the order β1‐β2‐β3‐α1‐α2‐α3‐α4‐β4‐α5‐α6‐β5‐α7‐α8‐α9. The five‐stranded β‐sheet forms the core of the protein. It is aligned in the order β3‐β2‐β1‐β4‐β5. Strand β2 is antiparallel to the rest. The β‐sheet is flanked by the α‐helices (Fig. 6A,B). Each monomer of MG Orn contains an Mn^2+^ ion in the active site, most probably resulting from buffers used for protein purification and storage of MG Orn. The metal ion is coordinated by Asp12, Glu14, both located on β1, and Asp163, located on α8 (Fig. 6B, Fig. S1).

**Table 1:** Data collection, processing, and structure refinement statistics. Values in parentheses are for the outermost shell

Diffraction source	BESSY II, BL 14.1
Wavelength (Å)	0.91841
Temperature (K)	100
Detector	PILATUS
Crystal‐to‐detector distance (mm)	647.31
Rotation range pr. image (°)	0.1
Total rotation range (°)	94
Space group	*P*3_1_21
*a*,* b*,* c* (Å)	108.32, 108.32, 101.33
α, β, γ (°)	90, 90, 120
Mosaicity (°)	0.16
Resolution range (Å)	42.57–3.15 (3.37–3.15)
Total no. of reflections	63 171 (11 289)
No. of unique reflections	12 264 (2200)
Completeness (%)	99.9 (100.0)
Multiplicity	5.2 (5.1)
<*Ι*/σ(*Ι*)>	8.2 (1.6)
*R*_p.i.m._	0.076 (0.551)
Overall *B* factor from Wilson plot (Å^2^)	65.44
σ cutoff	None
Final *R*_cryst_	0.2454
Final *R*_free_	0.2655
Rotamer outliers (%)	5.34
Clashscore	5.26
No. of non‐H atoms	
Protein	4376
Mn	3
Total	4379
R.m.s. deviations	
Bonds (Å)	0.003
Angles (°)	0.70
Average *B* factors (Å^2^)	
Overall	82.53
Protein	82.51
Mn	103.38
Ramachandran plot (%)	
Preferred	98.29
Allowed	1.71

A surface representation clearly shows a cavity within the protein comprising of the amino acid residues forming the active site and known as the DEDDh motif, that is, Asp12, Glu14, Asp112, Asp163, and His158 (Figs S1 and S2).

**Figure 7:**
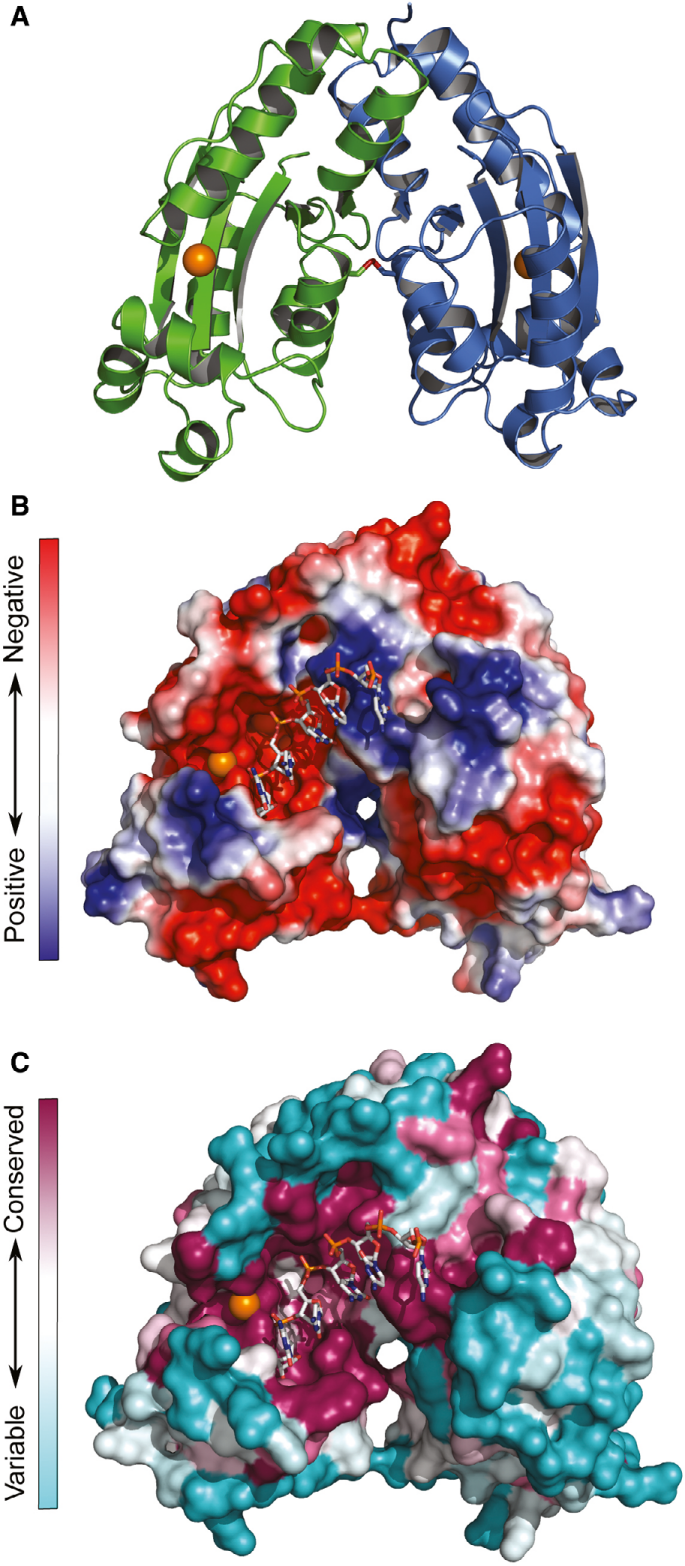
The functional dimer of MG Orn. (A) Cartoon representation of the functional dimer of MG Orn with the two monomers colored individually. The Mn^2+^ ion is indicated as an orange sphere, and the intermolecular disulfide bond is shown as sticks with sulfur atoms colored orange. (B) Electrostatic potential mapped onto the molecular surface of the MG Orn dimer. The colors range from red (negative potential) to blue (positive potential). (C) Sequence conservation from the ConSurf analysis mapped onto the molecular surface of the MG Orn dimer. The colors range from deep purple (conserved residues) to mint (variable residues). In B and C, the modeled 5mer RNA is shown as a stick model.

The three‐dimensional structure of MG Orn clearly indicates that monomer A and monomer C form a homodimer within the asymmetric unit, while monomer B forms a homodimer with a crystallographic copy of itself. The A–C homodimer is illustrated and focused on in the following discussion (Fig. 7A). The homodimers are connected through an intermolecular disulfide bond (representative electron density shown in Fig. S3). The dimer is further stabilized through salt bridges, H‐bonds, and hydrophobic interactions. The active site of each monomer, including coordination of the Mn^2+^ ion, is still accessible and exposed to the solvent in the homodimer.

A structural analysis through PDBePISA 17 highlighted the disulfide bond (C:Cys110 – A:Cys110), two salt bridges (C:Arg130 [NH1] – A:Glu139 [OE1] and C:Glu139 [OE1] – A:Arg130 [NH1]), and a total of 17 H‐bonds as important contributors to the formation of the stable homodimer. Furthermore, there are numerous hydrophobic interactions to stabilize the dimer interface, as a total of 20 amino acid residues from each monomer have more than 50% of their total area toward the interface. This accounts mainly to residues in the β5‐α7 region (MG Orn residues 130–145) as well as both termini.

However, several Orn sequences do not contain a Cys residue but an Ala and Gly residue at position 110, respectively (Fig. S2). Table 2 displays putative interactions adding to the formation of the dimer for the different Orn macromolecules. Of the compared structures, MG Orn has the smallest buried surface area and number of hydrophobic contributors upon dimer formation, while differences in number of H‐bonds and salt bridges are less pronounced. Interestingly, only some Orn proteins form an intermolecular disulfide bridge (Cys110‐Cys110′) crosslinking the monomers in the homodimer believed to be a major contributor to the overall stability of the homodimer. The effect of mutating the Cys residue in MG Orn was thus further investigated.

**Table 2:** Interface analysis of the MG Orn homodimer and its homologues. Performed with PDBePISA 17

Orn	PDB code	Buried area, Å^2^	aa residue at position 110	Number of disulfide bridges	Number of H‐bonds	Number of salt bridges	Number of hydrophobic contributors^a^
MG Orn	6RK6	1402.1	Cys	1	17	2	20
*Xanthomonas campestris* Orn	2GBZ	1753.7	Cys	1	19	4	28
*Coxiella burnetii* Orn	3TR8	1622.8	Cys	1	19	1	29
*Acinetobacter baumannii* Orn	5CY4	1602.0	Cys	1	18	6	24
*Haemophilus influenzae* Orn	1J9A	1638.4	Ala	0	21	4	27
*Escherichia coli* Orn	2IGI	1608.5	Gly	0	17	2	28
*Colwellia psychrerythraea* Orn	6A4A	1516.9	Gly	0	14	4	27

^a^Hydrophobic residues with more than 50% of their total area toward the interface according to PDBePISA.

### Structural aspects of RNA substrate binding in MG Orn

**Figure 8:**
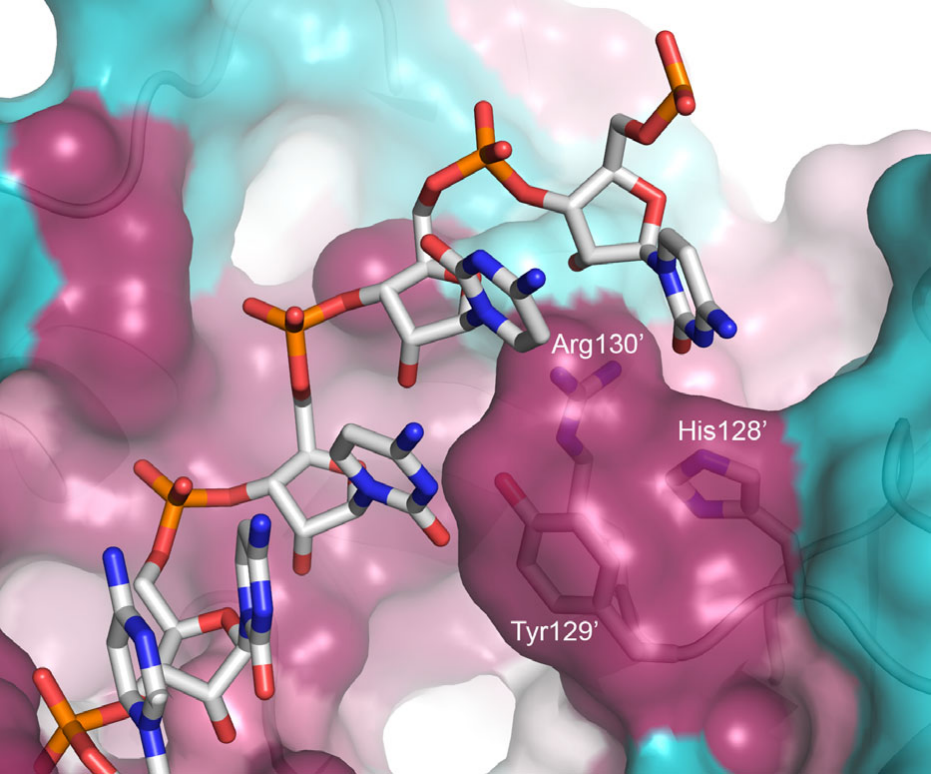
The 5′ end of the modeled 5mer RNA molecule is in proximity to the conserved sequence patch His128′, Tyr129′, and Arg130′ in the neighboring monomer. The surface is colored based on the ConSurf output with colors ranging from deep purple (conserved residues) to mint (variable residues).

In order to investigate the structural basis for the observed *in vitro* processing of longer substrates (5mer, 7mer, and 10mer RNA) shown for MG Orn, we used complexed *E. coli* exonuclease I (ExoI) as a model. ExoI is a three‐domain protein, where the N‐terminal domain has homology to the DnaQ superfamily. The crystal structure of ExoI in complex with ssDNA was superpositioned to the MG Orn structure (sequence identity of 13.2% for 151 aligned amino acid residues) with an r.m.s. deviation of 2.33 Å. The overlaid structure formed the template for manual fitting of a 5mer RNA molecule into the substrate binding cleft and active site of MG Orn. The modeled 5mer RNA molecule was visualized onto both the electrostatic potential and ConSurf molecular surfaces of the functional dimer of MG Orn (Fig. 7B,C). Nucleotides in the 5′ end of this model appear to be in tight interaction with a conserved sequence patch (His128′‐Tyr129′‐Arg130′) in the second monomer in the functional homodimer of MG Orn (Fig. 8). This sequence patch is most likely of importance for interaction with longer substrates.

### Role of the intermolecular disulfide bond for homodimer formation and nuclease function

The importance of the intermolecular disulfide bond connecting two Orn monomers was demonstrated by comparing the biochemical properties of MG Orn and the two variants OrnC110G and OrnC110A. Following the same procedure as for MG Orn production, OrnC110A and OrnC110G were recombinantly produced in *E. coli* and purified to homogeneity (Fig. S4).

Thermal stability of MG Orn, OrnC110A, and OrnC110G was evaluated by ThermoFluor assay, monitoring changes in hydrophobic fluorescent dye binding upon protein unfolding. All proteins followed the expected shapes of a thermal denaturation profile, displaying an observable melting transition between folded and unfolded states. MG Orn showed a broader thermal unfolding profile compared to the narrower profile of OrnC110A and OrnC110G, possibly reflecting two transitions of the dimeric MG Orn (Fig. S4). The calculated melting temperature (*T*_m_) for the three enzyme variants was similar being 55 °C for MG Orn and 55.6 °C and 54.7 °C for OrnC110A and OrnC110G, respectively (Fig. S4).

**Figure 9:**
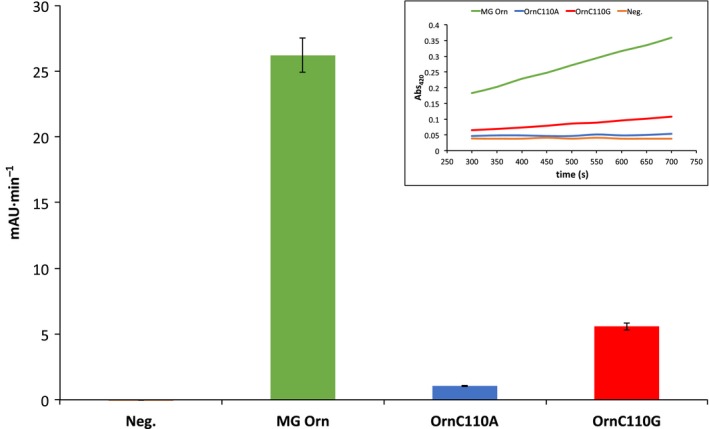
Enzymatic activity of MG Orn (green), OrnC110A (blue), and OrnC110G (red). RNA degradation was tested with the time‐resolved *p*NP‐TMP activity assay with 1.5 mm *p*NP‐TMP and 2 μg Orn in 50 mm Tris pH 8, 200 mm NaCl, 1 mm MnCl_2_ at 25 °C. The graph shows the increase in absorbance over time for MG Orn, its mutants, and the negative control (Neg.). The calculated error bars denote the standard deviation between duplicate runs. The inset shows the absorbance (Abs_420_) of the reaction product plotted against the time for each enzymatic reaction.

Two different assay setups were used to investigate the effect of mutating the intermolecular disulfide bridge in the homodimer. The first assay was monitoring the nuclease activity using *p*NP‐TMP, a dinucleotide mimic of a natural nucleic acid, as substrate. The C110G mutation abolished approximately 80% of enzymatic activity on *p*NP‐TMP, compared to MG Orn, whereas the C110A mutation almost completely inactivated the enzyme (Fig. 9).

**Figure 10:**
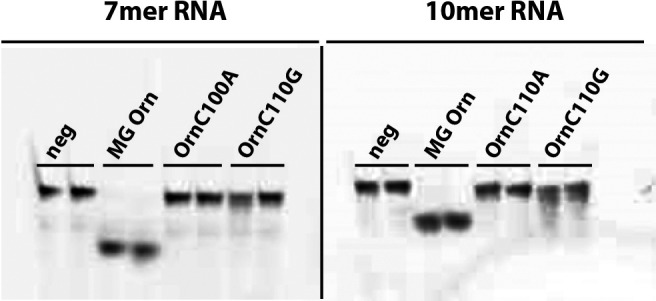
Nuclease activity of MG Orn and variants on 7mer and 10mer RNA substrates. RNA degradation was investigated using the gel‐based nuclease activity assay with 25 nm substrate and 0.8 μg enzyme in 50 mm Tris pH 8.0, 200 mm NaCl, 0.2 mg·mL^−1^ BSA, 2% glycerol, and 1 mm MnCl_2_ for 15 min at 25 °C. Samples were analyzed on 20% denaturing PAA gels (8 × 8 cm). Reaction buffer replaced protein solution in the negative control (Neg).

The second assay employed utilized RNA molecules of different length as substrate. While MG Orn displayed a robust exoribonuclease activity on both 7mer and 10mer RNA, OrnC110A showed complete loss of activity on these RNA substrates. The degradation pattern indicates that OrnC110G may have a miniscule capacity to act on both 7mer and 10mer (Fig. 10). Similar results were obtained by increasing the reaction temperature to 37 °C (Fig. S5).

**Figure 11:**
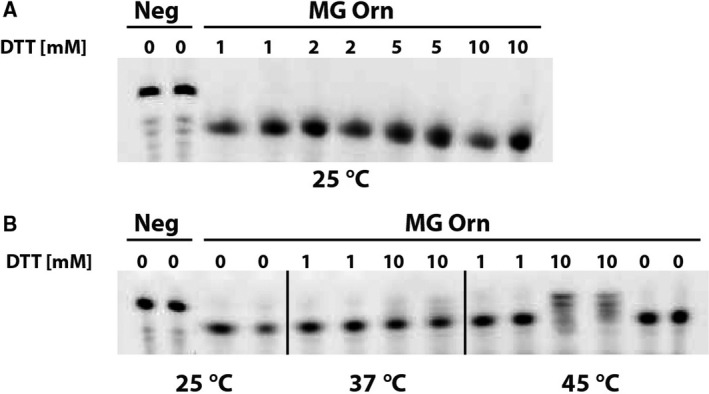
(A, B) Effect of DTT and temperature on the nuclease activity of MG Orn. Reactions were performed using the gel‐based nuclease activity assay with 25 nm 7mer RNA substrate and 0.8 μg MG Orn in 50 mm Tris pH 8.0, 200 mm NaCl, 0.2 mg·mL^−1^ BSA, 2% glycerol, 1 mm MnCl_2_ with various concentrations of DTT as indicated in A and B at different temperatures (25 °C, 37 °C, and 45 °C) for 15 min and analyzed on 20% denaturing PAA gels (8 × 8 cm).

In order to investigate the role of the disulfide bridge after forming the functional dimer, we added increasing amount of the reducing agent DTT to MG Orn at three different temperatures (Fig. 11A,B). MG Orn showed activity in the presence of up to 10 mm DTT at 25 °C and 37 °C. Partial inhibition of activity could be detected only in the presence of 10 mm DTT at 45 °C.

## Discussion

Phylogenetic analysis of the Svalbard metagenome Orn (MG Orn) described here strongly indicates that MG Orn originates from a species within the *Arenicellales* order, possibly *Arenicellas* or another close relative *Ar. xantha*. Although the dataset is limited (175 residues), the close relation of MG Orn to *Ar. xantha* Orn and its identification as a gammaproteobacterium is strongly supported by this result.

MG Orn is shown to exist as a dimer in solution in correspondence with other described Orn enzymes such as *E. coli* Orn 12, 13. It has a 3′–5′ directionality and rapidly degrades small oligoribonucleotides to monoribonucleotides. MG Orn prefers Mn^2+^ over Mg^2+^ as divalent cation at pH 8.5 for optimal nuclease activity and possesses a quite broad salt tolerance. This broad salt tolerance, with maximum approximately between 250 and 500 mm NaCl and detectable activity up to 2 m, may arise due to its marine origin and the variable salt tolerance in the littoral zone 18. As expected, originating from a cold marine habitat MG Orn shows significantly lower thermal stability compared to its mesophilic *E. coli* counterpart. MG Orn showed a *t*_1/2_ of 15 min at 50 °C, while *E. coli* Orn previously has been shown to still retain 50% residual activity after 60 min at 65 °C 11.

The three‐dimensional structure of MG Orn indicates that it functions as a homodimer, where two monomers are connected to each other through an intermolecular disulfide bond (Fig. S3). In addition, several other interactions also contribute to the dimerization interface including hydrophobic interactions, salt bridges, and H‐bonds.

In this study, we wanted to investigate the functional role of the intermolecular disulfide bridge (Cys110‐Cys110′) connecting the two monomers, and mutated Cys110 to Gly and Ala, amino acid residues naturally occurring at the respective position in other deposited Orn structures. The thermal stability of MG Orn, OrnC110A, and OrnC110G was investigated using a ThermoFluor assay. The ThermoFluor data show a broader thermal unfolding profile for MG Orn compared to OrnC110A and OrnC110G, possibly reflecting two transitions of the dimeric MG Orn. This broader profile is probably due to dissociation of the dimer ahead of monomer unfolding. Dimer disruption allows access of the fluorescence dye to the revealed hydrophobic areas of the interface, leading to an earlier increase in the recorded fluorescence intensity. Thus, the thermal unfolding temperature of MG Orn and its variants is around 55 °C, indicating that mutation of the cysteine involved in dimer formation does not influence the thermal stability of Orn.

Using the dinucleotide substrate mimic *p*NP‐TMP, as well as oligoribonucleotides of different lengths, we could show that the mutations severely affected MG Orn's ability to act as an exoribonuclease. These results indicate that residue C110 and its intermolecular disulfide bond are essential for homodimer formation and catalytic function of MG Orn. However, other interactions must also be important for maintaining the dimer formation once it is formed and was further proven by adding the reducing agent DTT to MG Orn. MG Orn showed surprising resilience toward DTT, and inhibition of exoribonuclease activity could only be detected using 10 mm DTT at 45 °C.

Coordination of the Mn^2+^ ion by Asp12 and Glu14, located on β1, and Asp163, located on α8, is also seen by the corresponding amino acid residues in *Cox. burnetii* Orn (PDB code 3TR8). *X. campestris* Orn and *Colwellia psychrerythraea* Orn (PDB code 2GBZ and 6A4A, respectively), on the other hand, each contain one Mg^2+^ ion. The site for binding of Mg^2+^ differs slightly from the binding site for Mn^2+^. However, in both protein structures the Mg^2+^ ion is coordinated by Asp12 and Glu14 as is the Mn^2+^ in MG Orn and *Cox. burnetii* Orn. Whereas Glu14 shows the same orientation in all four proteins, the orientation of Asp12 depends on the nature of the metal‐ion bound that is tilted by 35°. Binding of Mg^2+^ in *X. campestris* Orn and *Col. psychrerythraea* Orn is further supported by Asp112, residing on α5. The respective Asp residue in MG Orn and *Cox. burnetii* Orn is not involved in binding of the Mn^2+^ ion. Asp112, involved in Mn^2+^ binding as mentioned above, is also involved in Mg^2+^ binding in *Col. psychrerythraea*. Compared to Asp163 in MG Orn, this Asp residue is tilted toward the metal ion by ~ 40°.

Lately, a paper describing binding of U‐U and *p*NP‐TMP has been published 16. In our study, we have shown that MG Orn efficiently acts on the dinucleotide analogue *p*NP‐TMP as well as on 5mer, 7mer, and 10mer RNA substrates. In order to explain the structural basis for the observed *in vitro* processing of ‘longer’ oligoribonucleotides shown for MG Orn, complexed *E. coli* exonuclease I (ExoI) was used as a template for manual docking of 5mer RNA into the binding pocket. Although the sequence identity between MG Orn and ExoI is low, the structure‐based alignment revealed interesting conservation in the active‐site region. Notably, except for His158 (which appears to be in a somewhat flipped‐out state in the MG Orn structure), all amino acid residues in the signature DEDDh cluster were structurally conserved between the two structures.

The coordination of the 3′ end of the nucleotide substrate (ssDNA in ExoI; RNA in MG Orn) into the respective active sites is at overlapping positions in MG Orn compared to ExoI. There is a marked difference in polarity between ExoI and MG Orn in the region around the 2′‐position of the 3′‐sugar unit of the oligonucleotide. Where this area is relatively spacious and nonpolar in ExoI, corresponding to the nature of the deoxyribose in an ssDNA substrate, it is instead rather polar in MG Orn (Thr and Ala in ExoI are replaced with His and Asn in MG Orn). A loop region (His128′, Tyr129′, and Arg130′) from the neighboring monomer in the functional dimer is in proximity to the 5′ region of the modeled RNA molecule. Tyr129′ and Arg130′ have very recently been implicated as important for binding and processing of dinucleotides. When Tyr129′ was exchanged to Ala in *Col. psychrerythraea* Orn, no significant change in hydrolytic activity against *p*NP‐TMP was observed, thus indicating that Tyr129′ does not play a vital role in processing of dinucleotide substrates 16. However, based on modeling of MG Orn with a 5mer RNA, there are clear indications that these residues indeed play an important role in stabilizing the RNA substrate when MG Orn is processing RNA molecules longer than dinucleotides. The importance of these residues is further supported by the fact that the residues in this loop are completely conserved among 150 Orn homologues.

## Conclusion

This study highlights the importance of dimer formation for substrate binding and subsequent catalytic action in MG Orn. We show an important role of an intermolecular disulfide bond for the formation of the homodimer, which proves to be essential for the ability of the enzyme to degrade small oligoribonucleotides. We also show the *in vitro* ability of MG Orn to act on ‘longer’ RNA oligos (5–10mer), probably through the involvement of a conserved sequence loop (His128′, Tyr129′ and Arg130′) in the neighboring monomer when binding longer RNA substrates.

## Methods

### Bioinformatics

A maximum likelihood (ML) phylogeny (JTT model) was constructed based on a dataset containing nine Orn sequences, seven from gammaproteobacteria and two from betaproteobacteria (used as outgroup), using the mega x software 19, 20. A bootstrap analysis was done to test the stability of nodes, using the ML method and JTT model, with 500 pseudoreplicates 19. The protein alignment included sequences from *Ar. xantha* WP_113955167.1, *X. campestris* WP_011037314.1, *P. aeruginosa* RQB22498.1, *Cox. burnetii* WP_005770781.1, *Ac. baumannii* SST03775.1, *H. influenzae* WP_021035403.1, *E. coli* WP_042004351.1, *Bordetella trematum* WP_025512385.1, and *Achromobacter piechaudii* WP_006218241.1. Alignment files were generated using clustalw 21.

### Cloning of the gene encoding MG Orn

The *mg orn* gene (Fig. S6) has been cloned into the pENTR™/TEV/D‐TOPO™ entry vector by Directional TOPO^®^ Cloning from Thermo Fisher Scientific (Waltham, MA, USA) (forward primer: 5′‐CACC GTG CCG CAA AAC CCA AAT GTT‐3′, reverse primer: 5′‐ TTA GTT CAT ATC GAG CAG TAT CAG ATT GTT TCG‐3′). Positive clones have been confirmed by sequencing analysis. The gene has been subsequently transferred into the destination vector pHMGWA by the LR Clonase reaction using Gateway™ LR Clonase™ II Enzyme Mix (Thermo Fisher Scientific). Positive clones have been confirmed by sequencing analysis. Due to the cloning procedure applied, the *mg orn* gene could be expressed with an N‐terminal His_6_‐MBP‐tag followed by a recognition sequence for the TEV protease (TEV protease).

### Preparation of mutant constructs

Substitution of Cys110 by Ala and Gly, respectively, was performed using the QuikChange II Site‐Directed Mutagenesis Kit (Agilent Technologies, Santa Clara, CA, USA). The pHMGWA plasmid containing *mg orn* was used as a template for single substitutions with synthetic oligonucleotide primers (OrnC110A: forward primer: 5′‐GCG GTA ATA GCA TTG CGC AAG ATC GCC G‐3′, reverse primer: 5′‐CGG CGA TCT TGC GCA ATG CTA TTA CCG C‐3′; OrnC110G: forward primer: 5′‐GCG GTA ATA GCA TTG GCC AAG ATC GCC G‐3′, reverse primer: 5′‐CGG CGA TCT TGG CCA ATG CTA TTA CCG C‐3′). Both mutations were confirmed by sequencing analysis.

### Recombinant expression

For recombinant expression of *mg orn* with an N‐terminal His_6_‐MBP‐tag, the plasmid has been transformed into Rosetta 2 (DE3) cells (Merck KGaA, Darmstadt, Germany). Several colonies were picked and incubated in 50 mL LB media containing 100 μg·mL^−1^ ampicillin at 37 °C, 225 r.p.m., overnight. One liter of LB/ampicillin (100 μg·mL^−1^) medium was inoculated with 20 mL of overnight culture and grown at 37 °C, 180 r.p.m., until cell density reached OD_600_ of 0.5. Gene expression was induced by addition of 0.5 mm IPTG, and protein production was carried out for 4 h at 20 °C and 180 r.p.m.

### Protein purification of MG Orn, OrnC110A, and OrnC110G

Cell lysis and all purification steps were carried out at 4 °C. Cell pellets from a 1‐L cultivation were resuspended in 30 mL lysis buffer [50 mm Tris pH 7.4 (at 25 °C), 300 mm NaCl, 1 mm MnCl_2_], one protease inhibitor tablet (cOmplete™, Mini, EDTA‐free Protease Inhibitor Cocktail; Roche, Basel, Switzerland), and 10 μg·mL^−1^ lysozyme. After incubation on ice for 30 min, cell lysis was performed by sonication with the VCX 750 from Sonics^®^ (Newtown, CT, USA) (pulse 1.0/1.0, 20 min, amplitude 25%). Insoluble cell debris was removed by centrifugation (48 384 ***g***, 45 min, 4 °C). The supernatant was filtered through a 0.45‐μm membrane and loaded onto a 1‐mL HisTrap™ HP column (GE Healthcare, Wood Dale, IL, USA) in buffer A [50 mm Tris pH 7.4 (at 25 °C), 300 mm NaCl, 1 mm MnCl_2_] and washed in three steps: (a) buffer A2 [50 mm Tris pH 7.4 (at 25 °C), 1 m NaCl, 1 mm MnCl_2_], (b) buffer A, and (c) 5% buffer B [50 mm Tris pH 7.4 (at 25 °C), 300 mm NaCl, 1 mm MnCl_2_, 500 mm imidazole]. His_6_‐MBP‐tagged protein was eluted on a 20 mL gradient from 5% to 100% buffer B. Fractions containing the protein were exchanged into buffer A by using a 5‐mL HiTrap™ Desalting column (GE Healthcare). TEV protease cleavage was performed overnight with 0.1 mg TEV per 1 mL of eluted fusion protein in 50 mm Tris pH 8.0 (at 25 °C), 0.5 mm EDTA, and 1 mm DTT at 4 °C (TEV protease produced in‐house). Protease‐treated protein was separated from remaining impurities and the His_6_‐MBP‐tag by reverse affinity chromatography on a 1‐mL HisTrap™ HP column with buffer A. Fractions from flow‐through and the initial phase of the first gradient at 5% buffer B were collected, added to an equal amount of buffer A, and loaded onto a 1 mL HiTrap™ Blue HP column (GE Healthcare) in buffer A3 [50 mm HEPES pH 7.4 (at 25 °C), 50 mm NaCl, 1 mm MnCl_2_]. The protein was eluted using 100% buffer B2 [50 mm HEPES pH 7.4 (at 25 °C), 2 m NaCl, 1 mm MnCl_2_], and eluted peak fractions were analyzed by SDS/PAGE. Fractions containing the protein were exchanged into buffer C [20 mm HEPES pH 7.4 (at 25 °C), 150 mm NaCl, 1 mm MnCl_2_], up‐concentrated to approximately 1 mg·mL^−1^ using Amicon Ultra centrifugal filter units (MWCO 10 kDa, Merck KGaA), and stored at −20 °C for activity and stability assays with 50% (v/v) glycerol.

### Size‐exclusion chromatography

For crystallization trials and characterization of MG Orn stoichiometry, size‐exclusion chromatography was performed. Up‐concentrated Orn eluted from the HiTrap™ Blue HP column was run on a HiLoad^®^ 16/600 Superdex^®^ 200 pg column (GE Healthcare) at 1 mL·min^−1^ with 20 mm HEPES pH 7.5 (at 25 °C), 150 mm NaCl, 1 mm MnCl_2_. Fractions containing MG Orn were collected and concentrated up to 3.6 mg·mL^−1^ for crystallization trials. For molecular weight determination, and thus stoichiometry, standard proteins [Ferritin (440 kDa), Conalbumin (75 kDa), Ovalbumin (43 kDa), Carbonic Anhydrase (29 kDa), RNase A (14 kDa), and Aprotinin (6.5 kDa)] have been applied onto the HiLoad^®^ 16/600 Superdex^®^ 200 pg column (GE Healthcare) at the same flow rate and buffer as mentioned for MG Orn. The known molecular weight and the elution volume of the individual proteins have been used to draw up a calibration curve. Based on this calibration curve and its elution volume, the molecular weight for the MG Orn macromolecule in solution has been calculated.

### ThermoFluor assay

The melting temperature (*T*_m_) of Mg Orn, OrnC110A, and OrnC110G, thus the thermal stability of the proteins, was determined by ThermoFluor experiments according to Ref. 22. The reactions contained 50 mm HEPES pH 7.5 (at 25 °C), 72 mm NaCl, SYPRO^®^ Orange (Merck KGaA) in a final dilution of 6× and 4 μg of protein. All components were mixed thoroughly in a well of a thin‐wall PCR plate (Bio‐Rad, Hercules, CA, USA). The wells were sealed with optical‐quality sealing tape (Bio‐Rad). The volume of the final reaction was 25 μL. A temperature range of 10–90 °C with an increment of 0.3 °C at 3‐s intervals has been scanned in the ThermoFluor experiment (excitation at 495 nm, emission at 556 nm).

### Protein crystallization and X‐ray data collection

Crystallization experiments were performed with a stock solution of purified MG Orn at 3.6 mg·mL^−1^ in 20 mm HEPES pH 7.5 (at 25 °C), 150 mm NaCl, 1 mm MnCl_2_. Initial crystallization conditions were screened using the vapor diffusion method set up by a Phoenix crystallization robot (Art Robbins Instruments, Sunnyvale, CA, USA). The plates were set up with 60 μL reservoir solution and sitting drops with equal amounts of reservoir solution mixed with protein stock solution in a total drop volume of 0.5 μL. The screens were incubated at 4 °C. Diffraction‐quality crystals were found after 2 weeks at a condition containing 0.1 m HEPES pH 7 (at 25 °C) and 5% PEG 8000. Crystals were harvested, transferred through a cryoprotectant solution consisting of the reservoir solution with 30% (v/v) glycerol added, and flash‐cooled in liquid N_2_. X‐ray diffraction data were collected at BL14.1 operated by the Helmholtz‐Zentrum Berlin (HZB) at the BESSY II electron‐storage ring (Berlin‐Adlershof, Germany; Ref.^23^). The data were indexed and integrated by xds/xscale 24, before being merged and scaled by programs in the ccp4 program suite 25. Data collection and processing statistics are presented in Table 1.

### Structure determination, refinement, and analysis

The crystal structure was determined by molecular replacement using phaser 26 in the phenix program package 27 with one monomer of Orn from *X. campestris* (PDB 2GBZ; Ref.^15^) as initial search model. autobuild 28 traced the full length of one of the three monomers in the asymmetric unit, and this was subsequently fed back into phaser, resulting in improved map quality. The manual model building was done in coot 29 interspersed by cycles of refinement using phenix.refine 30 and converged at final *R*_cryst_/*R*_free_ values of 24.54/26.55. A summary of the refinement statistics is shown in Table 1. The atomic coordinates and structure factors have been deposited in the RCSB Protein Data Bank (www.rcsb.org) with the accession code 6RK6. Figures presented in Results section were generated using pymol (pymol.org).

The crystal structure of *E. coli* Exonuclease I (ExoI) in complex with ssDNA (PDB code 4JRP) was superpositioned onto the crystal structure of MG Orn using wincoot 29. Based on the corresponding bound ssDNA fragment in ExoI, a 5mer RNA fragment (CCCCC) in the A‐form was generated in WinCoot and manually fitted to the MG Orn active site. Shape and charge complementarities were taken into account in the adjustment. Electrostatic surface potentials were calculated through the APBS 31 plugin in pymol (The PyMOL Molecular Graphics System, Version 2.0 Schrödinger, LLC). Mapping of conserved amino acids onto the molecular surface of a dimer of MG Orn was performed through the ConSurf server 32, 33. Default parameters were used, selecting 150 sequences with sequence identities in the 35–95% range. The output was visualized using pymol.

### pNP‐TMP activity assay

The time‐resolved *p*NP‐TMP activity assay was performed according to Hamdan *et al*. 34. In Falcon^®^ 96‐well assay plates, up to 2 μg Orn was mixed with reaction buffer [50 mm Tris pH 8 (at 25 °C), 200 mm NaCl, 1 mm MnCl_2_] and 1.5 mm thymidine 5′‐monophosphate *p*‐nitrophenyl ester sodium salt (*p*NP‐TMP; Merck KGaA) in a total volume of 100 μL. The average change in absorption (420 nm) at 25 °C was measured by monitoring the initial 100 s of each reaction using a SpectraMax^®^ M2^e^ Microplate Reader (Molecular Devices, San Jose, CA, USA).

### Gel‐based nuclease activity assay

If not mentioned otherwise, ten microliter reactions contained 25 nm substrate (Table 3), 50 mm Tris pH 8.0 (at 25 °C), 200 mm NaCl, and 1 mm MnCl_2_. The enzyme was added in varying amounts as indicated with each figure. The enzymatic reaction took place at various incubation times and temperatures as indicated with each figure. Addition of 2.5 μL of denaturing gel loading buffer (95% formamide, 10 mm EDTA, 0.1% xylene cyanol) terminated the reaction after the desired incubation period. Samples were heated at 95 °C for 2 min. Six microliter of each sample was loaded onto denaturing polyacrylamide gels [12% or 20% polyacrylamide/7 m urea (denaturing PAA)], and gel electrophoresis was performed in 1× TBE buffer (89 mm Tris, 89 mm boric acid, 2 mm EDTA) at 50 W (40 × 20 cm PAA gels) or 180 V (8 × 8 cm PAA gels) for 1 h 15 min to 1 h 30 min.

**Table 3:** Sequences of RNA substrates employed in the gel‐based nuclease activity assay. [FAM], derivative of the fluorophore Fluorescein

RNA substrate	Sequence (5′–3′)	Investigation of
5mer	[FAM]CCCCC	Directionality
7mer	[FAM]CCCCCCC	Effect of salt
		Effect of mutation at position 110
		Effect of reducing agent and temperature
10mer	[FAM]CCCCCCCCCC	Effect of mutation at position 110

Distribution of the degradation products of the endpoint activity assay was monitored by scanning the gels for FAM fluorescence (excitation at 495 nm, emission at 517 nm) in a PharosFX Plus Imager (Bio‐Rad). Analysis of the gels was performed with quantity one 1‐d Analysis Software (Bio‐Rad).

### Enzyme assay for determination of directionality

Ten microliters of reaction contained 25 nm RNA substrate 7mer‐62OMe (5′‐[FAM]CCCCC[mC]C‐3′), 50 mm Tris pH 8.0 (at 25 °C), 150 mm NaCl, 1 mm MnCl_2_, 1 mm DTT, 0.2 mg·mL^−1^ BSA, and 2% glycerol. The reaction was started by addition of 0.74 μg protein and incubated at 25 °C for 2, 5, and 10 min. Reactions were stopped by addition of 2.5 μL denaturing gel loading buffer (95% formamide, 10 mm EDTA, 0.1% xylene cyanol) and incubation at 95 °C for 5 min. For the denaturing polyacrylamide gel electrophoresis (12% polyacrylamide/7 m urea, 40 × 20 cm) a sample volume of 6 μL was loaded onto the gel. Gel electrophoresis was performed in 0.5× TBE buffer (44.5 mm Tris, 44.5 mm boric acid, 1 mm EDTA) at 50 W for 1 h 15 min, and the gel was subsequently scanned for FAM‐fluorescence (excitation at 495 nm, emission at 517 nm) with the PharosFX Plus Imager (Bio‐Rad).

## Conflict of interest

The authors declare no conflict of interest.

## Author contributions

YP has been primarily responsible in planning the experiments, performed experiments such as testing the directionality of Mg Orn, analyzed data, and contributed to writing the paper. KB has been involved in planning and performed experiments such as mutagenesis of Mg Orn, protein production of OrnC110A and OrnC110G, and comparison studies of the mutants to the wild‐type enzyme. She contributed to writing the paper. DPK has been involved in planning and performed experiments such as cloning of Mg Orn, protein production, purification and crystallization of Mg Orn, and basic characterization thereof. IL has been responsible for three‐dimensional structure determination of Mg Orn, analysis thereof, and writing the paper. ANL had the original project idea, has been involved in planning experiments and analyzing data, and was primarily responsible for writing the paper.

## Supporting information


**Fig. S1.** Representative view of the coordination of the modelled Mn^2+^‐ion in each of the MG Orn monomers. Highlighted amino acids are shown as sticks in atom colours, while the rest of the protein is shown as a cartoon with colouring scheme as for Figure 6. Indicated distances are given in Å.
**Fig. S2.** Structure‐based sequence alignment of MG Orn with other determined structures of Orn homologs. The secondary structure elements of MG Orn are displayed in the top rows, where spirals and arrows depict α‐helix and β‐strands, respectively. Identical residues are shown in white on red background, while highly conserved residues are shown in red. Cys110 is indicated by a black asterisk, while residues in the conserved DEDDh motif are indicated by blue triangles. PDB identifiers: 2GBZ: *X. campestris* Orn; 3TR8: *C. burnetii* Orn; 5CY4: *A. baumannii* Orn; 1J9A: *H. influenzae* Orn; 2IGI: *E. coli* Orn; 6A4A: *C. psychrerythraea* Orn.
**Fig. S3.** Representative electron density displaying the region around the intermolecular disulphide bond connecting two MG Orn monomers. The electron density map is displayed at 1.3 times the r.m.s. deviation.
**Fig. S4.** Purification and thermal stability of MG Orn and its variants OrnC110A and OrnC110G. (A) SDS‐PAGE gel showing purified proteins after the final purification step (standard marker Novex Mark 12, Thermo Fisher Scientific). The grey arrow marks the position of the proteins at approximately 21.5 kDa. (B) Thermofluor experiments showing the melting curves of MG Orn (green), OrnC110A (blue) and OrnC110G (red) in 50 mM HEPES pH 7.5. The thermal unfolding was recorded from 10 °C to 90 °C, in increments of 0.3 °C per sec, and the fluorescence signal was plotted as a function of temperature. The table inset sums up the measured *T*m for MG Orn and its variants.
**Fig. S5.** Nuclease activity of MG Orn and mutants on RNA 7mer and 10mer. RNA degradation was carried out in reaction buffer (50 mM Tris‐HCl pH 8.0, 200 mM NaCl, 1 mg/ml BSA, 5 mM DTT, 10% glycerol, 1 mM MnCl2) for 15 minutes reaction at 25 °C and 37 °C and analyzed on 20% PAA gels (8 x 8 cm). Substrate concentration was 0.05 μM and enzyme concentration was 1.16 μM. Control reactions were run without Orn.
**Fig. S6.** Nucleotide sequence encoding MG Orn.

## Data Availability

Oligoribonuclease (EC 3.1.13.3), PDB code 6RK6.
