# Supplementary material for: Characterization of an intertidal zone metagenome oligoribonuclease and the role of the intermolecular disulfide bond for homodimer formation and nuclease activity
Source: FEBS Open Bio. 2019 Aug 31;9(10):1674–88. doi: 10.1002/2211-5463.12720 (PMC6768110; doi:10.1002/2211-5463.12720)
Supplement: Supplementary file 1 — Fig. S1. Representative view of the coordination of the modelled Mn2+‐ion in each of the MG Orn monomers. Highlighted amino acids are shown as sticks in atom colours, while the rest of the protein is shown as a cartoon with colouring scheme as for Figure 6. Indicated distances are given in Å. Fig. S2. Structure‐based sequence alignment of MG Orn with other determined structures of Orn homologs. The secondary structure elements of MG Orn are displayed in the top rows, where spirals and arrows depict α‐helix and β‐strands, respectively. Identical residues are shown in white on red background, while highly conserved residues are shown in red. Cys110 is indicated by a black asterisk, while residues in the conserved DEDDh motif are indicated by blue triangles. PDB identifiers: 2GBZ: X. campestris Orn; 3TR8: C. burnetii Orn; 5CY4: A. baumannii Orn; 1J9A: H. influenzae Orn; 2IGI: E. coli Orn; 6A4A: C. psychrerythraea Orn. Fig. S3. Representative electron density displaying the region around the intermolecular disulphide bond connecting two MG Orn monomers. The electron density map is displayed at 1.3 times the r.m.s. deviation. Fig. S4. Purification and thermal stability of MG Orn and its variants OrnC110A and OrnC110G. (A) SDS‐PAGE gel showing purified proteins after the final purification step (standard marker Novex Mark 12, Thermo Fisher Scientific). The grey arrow marks the position of the proteins at approximately 21.5 kDa. (B) Thermofluor experiments showing the melting curves of MG Orn (green), OrnC110A (blue) and OrnC110G (red) in 50 mM HEPES pH 7.5. The thermal unfolding was recorded from 10 °C to 90 °C, in increments of 0.3 °C per sec, and the fluorescence signal was plotted as a function of temperature. The table inset sums up the measured Tm for MG Orn and its variants. Fig. S5. Nuclease activity of MG Orn and mutants on RNA 7mer and 10mer. RNA degradation was carried out in reaction buffer (50 mM Tris‐HCl pH 8.0, 200 mM NaCl, 1 mg/ml BSA, 5 mM DTT, 10% glycero [file FEB4-9-1674-s001.pdf]

Supporting information

Supplementary Fig. 1

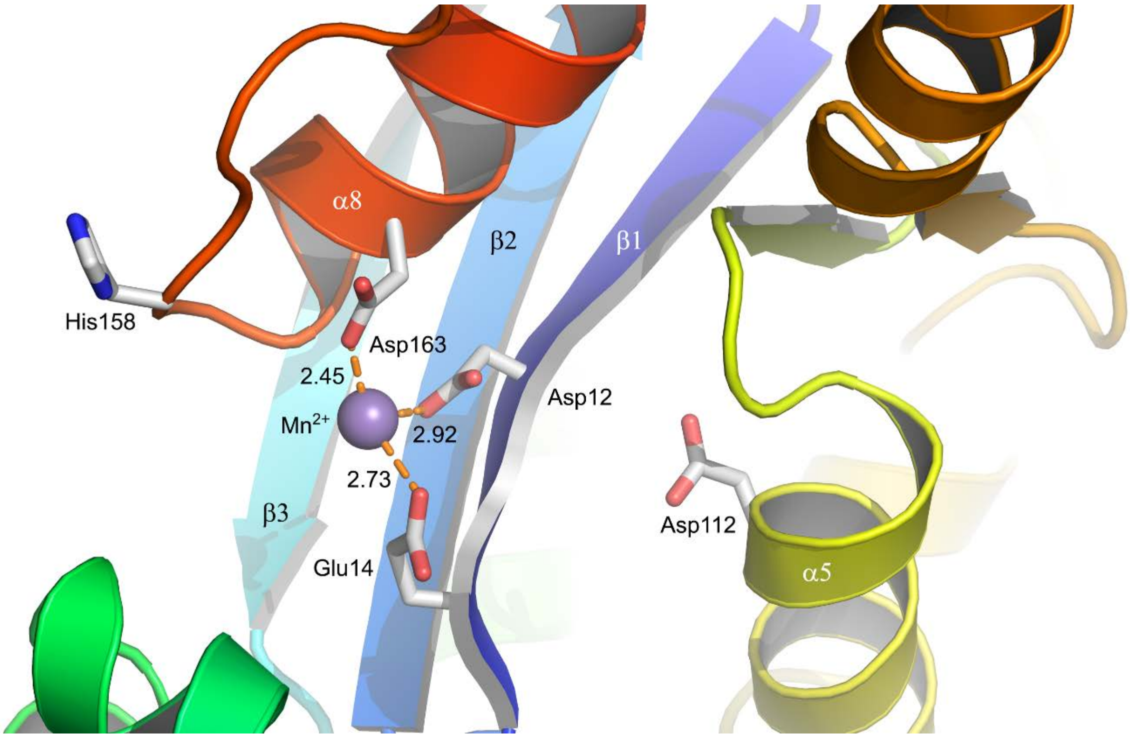

Supplementary Fig. 1. Representative view of the coordination of the modelled Mn^{2+}-ion in each of the MG Orn monomers. Highlighted amino acids are shown as sticks in atom colours, while the rest of the protein is shown as a cartoon with colouring scheme as for Figure 6. Indicated distances are given in Å.

Supplementary Fig. 2

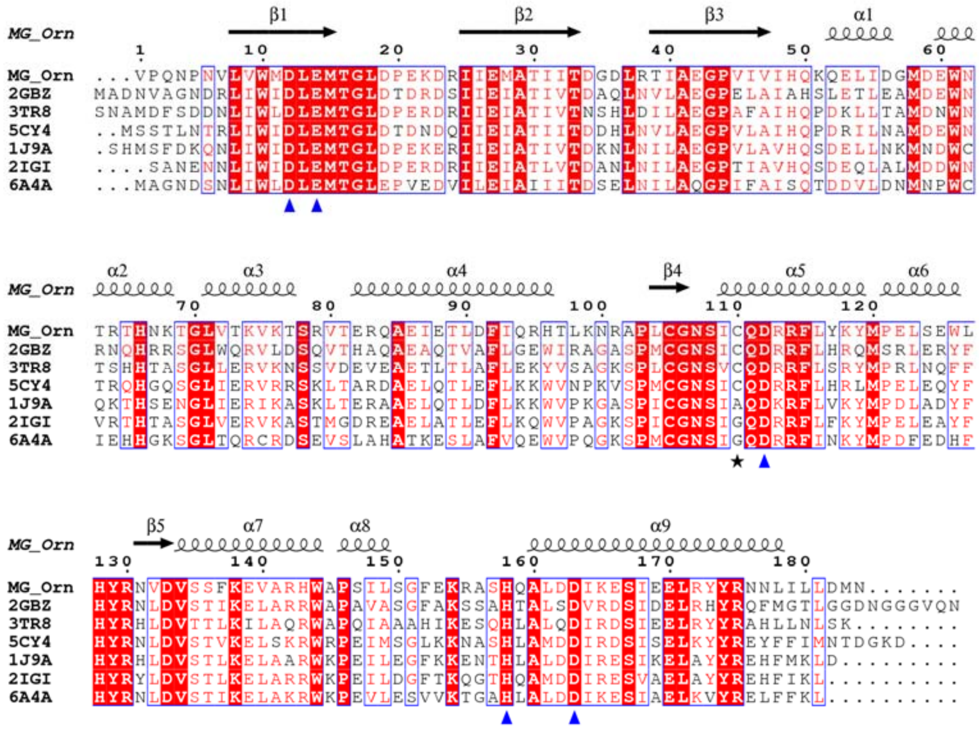

Supplementary Fig. 2. Structure-based sequence alignment of MG Orn with other determined structures of Orn homologs. The secondary structure elements of MG Orn are displayed in the top rows, where spirals and arrows depict α-helix and β-strands, respectively. Identical residues are shown in white on red background, while highly conserved residues are shown in red. Cys110 is indicated by a black asterisk, while residues in the conserved DEDDh motif are indicated by blue triangles. PDB identifiers: 2GBZ: *X. campestris* Orn; 3TR8: *C. burnetii* Orn; 5CY4: *A. baumannii* Orn; 1J9A: *H. influenzae* Orn; 2IGI: *E. coli* Orn; 6A4A: *C. psychrerythraea* Orn.

Supplementary Fig. 3

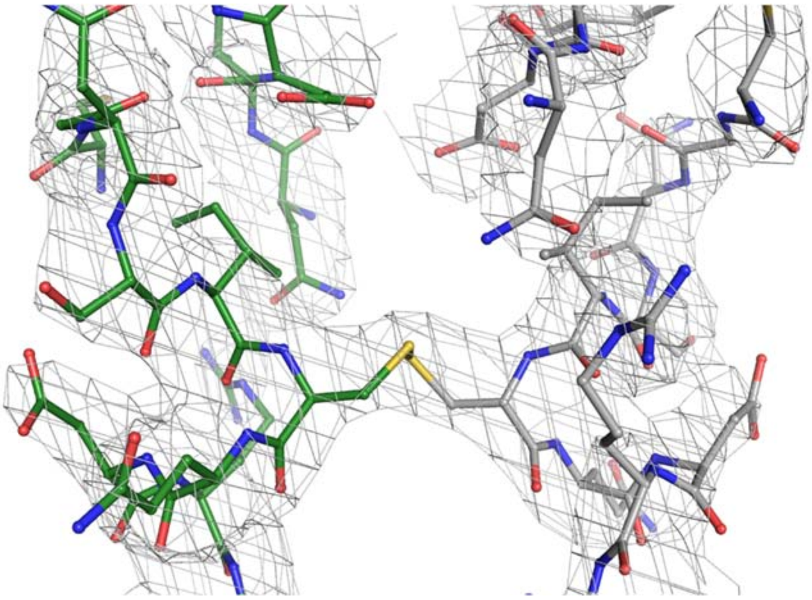

Supplementary Fig. 3. Representative electron density displaying the region around the intermolecular disulphide bond connecting two MG Orn monomers. The electron density map is displayed at 1.3 times the r.m.s. deviation.

Supplementary Fig. 4

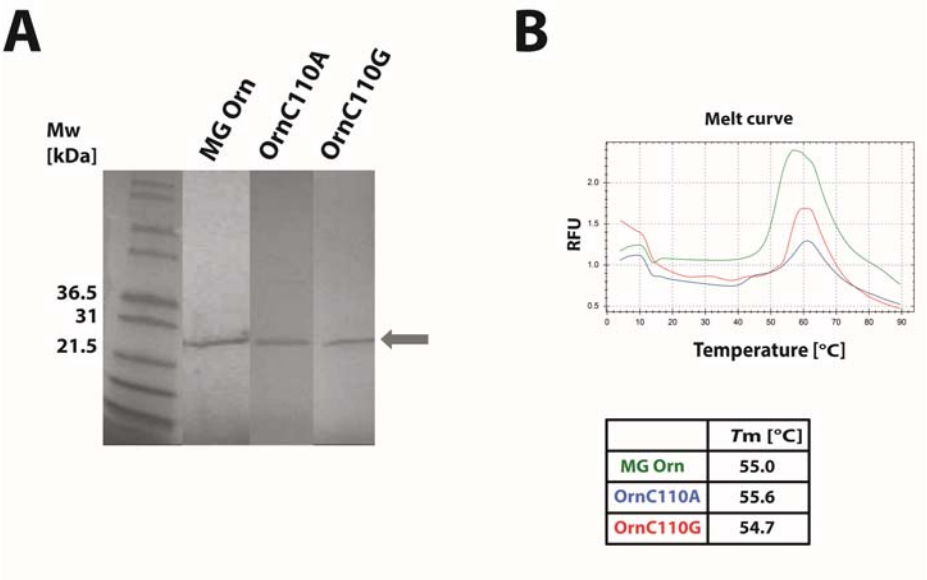

Supplementary Fig. 4. Purification and thermal stability of MG Orn and its variants OrnC110A and OrnC110G. (**A**) SDS-PAGE gel showing purified proteins after the final purification step (standard marker Novex Mark 12, Thermo Fisher Scientific). The grey arrow marks the position of the proteins at approximately 21.5 kDa. (**B**) Thermofluor experiments showing the melting curves of MG Orn (green), OrnC110A (blue) and OrnC110G (red) in 50 mM HEPES pH 7.5. The thermal unfolding was recorded from 10 ˚C to 90 ˚C, in increments of 0.3 ˚C per sec, and the fluorescence signal was plotted as a function of temperature. The table inset sums up the measured T_m for MG Orn and its variants.

Supplementary Fig. 5

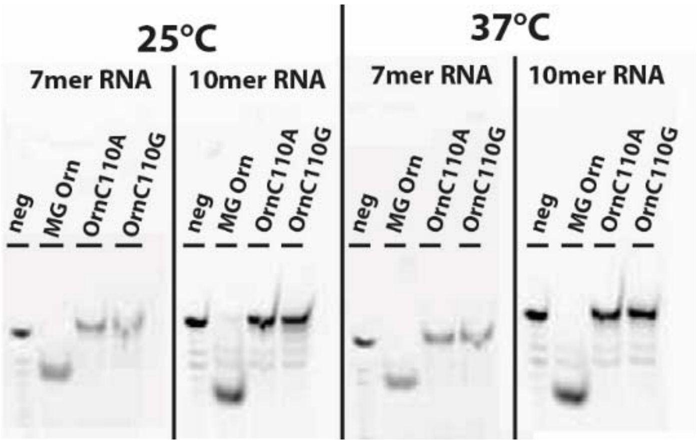

Supplementary Fig. 5. Nuclease activity of MG Orn and mutants on RNA 7mer and 10mer. RNA degradation was carried out in reaction buffer (50 mM Tris-HCl pH 8.0, 200 mM NaCl, 1 mg/ml BSA, 5 mM DTT, 10 % glycerol, 1 mM $MnCl_2$) for 15 minutes reaction at 25 ˚C and 37 ˚C and analyzed on 20 % PAA gels (8 x 8 cm). Substrate concentration was 0.05 µM and enzyme concentration was 1.16 µM. Control reactions were run without Orn.

Supplementary Fig. 6

GTGCCGCAAAACCCAAATGTTCTCGTCTGGATGGATCTCGAAATGACGGGGCTAG
ACCCAGAAAAAGACCGCATTATTGAAATGGCAACCATCATTACCGACGGCGATTT
GCGCACTATTGCTGAGGGGCCTGTGATTGTGATTCATCAAAAGCAAGAGCTCATC
GATGGAATGGACGAATGGAATACGCGTACCCATAACAAAACAGGTTTAGTCACT
AAGGTAAAAACCAGTCGTGTGACCGAACGTCAGGCCGAAATTGAAACTCTGGAT
TTTATTCAACGGCACACGCTCAAAAATCGCGCACCACTTTGCGGTAATAGCATTT
GCCAAGATCGCCGTTTTTTATACAAGTACATGCCTGAATTAAGCGAATGGCTGCA
TTATCGCAACGTAGATGTAAGCTCGTTTAAAGAAGTGGCCAGACATTGGGCGCCT
AGCATTCTCTCAGGTTTCGAAAAACGCGCATCGCATCAAGCTTTGGACGACATCA
AAGAATCTATTGAAGAGCTGCGTTACTACCGAAACAATCTGATACTGCTCGATAT
GAACTAA

Supplementary Fig. 6. Nucleotide sequence encoding MG Orn.
